# Closing the Gap: A Case Report on a Blood Patch Solution for Cerebrospinal Fluid Leak

**DOI:** 10.7759/cureus.50784

**Published:** 2023-12-19

**Authors:** Guilherme Sousa, Bárbara Alves, Filipa Cunha, Juliana Magalhães, Eduarda Figueiredo, Luís Abreu

**Affiliations:** 1 Anaesthesiology, Centro Hospitalar Tondela-Viseu, Viseu, PRT; 2 Neurology, Centro Hospitalar Tondela-Viseu, Viseu, PRT

**Keywords:** spontaneous intracranial hypotension (sih), epidural blood patch, refractory headache, regional anaesthesiology, spontaneous cerebrospinal fluid leak

## Abstract

Spontaneous intracranial hypotension is a condition resulting from cerebrospinal fluid leaks at the spinal level that disrupt the regulation of intracranial pressure. This disorder is an uncommon cause of debilitating headaches but can have variable clinical manifestations, which contributes to delayed diagnosis and potentially severe consequences. The standard treatment consists of conservative measures such as bed rest, hydration, and a pharmacological approach with paracetamol, caffeine, ergotamine, and dexamethasone. When conservative measures fail, an epidural blood patch is the gold standard treatment, where a small amount of blood is injected into the epidural space to form a clot to seal any existing leak. Recent studies showed a success rate of 64% without the need for further intervention.

The authors report a case of a 55-year-old woman with a three-month history of daily severe headaches. Imaging exams showed subdural collections, suggesting the hypothesis of cerebrospinal fluid hypotension. After the failure of conservative measures, an epidural blood patch was performed with progressive clinical improvement.

This case demonstrates the potential effectiveness of an epidural blood patch in the management of spontaneous intracranial hypotension and its complications, offering an encouraging option for those unresponsive to conservative measures. It also highlights the importance of a multidisciplinary approach involving neurologists and anesthesiologists.

## Introduction

Intracranial pressure is controlled by the production, flow, and absorption of cerebrospinal fluid (CSF). In cases of dysfunction of these mechanisms, a change in the CSF pressure will occur, which will probably lead to neurologic symptoms like nausea, vomiting, tinnitus, diplopia, and most commonly, headaches [1]. Spontaneous intracranial hypotension (SIH) is secondary to a CSF leak at the spinal level, which results in a loss of CSF volume that bathes the brain and spinal cord. The variability of signs and symptoms, along with a lack of awareness of the disorder, contributes to delayed diagnosis, which can have serious consequences. A common treatment (after the failure of conservative measures) is epidural patching with blood or fibrin sealant, which has good outcomes for most patients [1,2].

## Case presentation

We describe a case of a 55-year-old woman who presented with a three-month history of biparietal headache and cervical pain associated with auditory disturbances. The pain was exacerbated in the standing position and relieved in the supine position. There was no concomitant nausea or vomiting, a history of recent trauma, or lumbar puncture.

**Figure 1:**
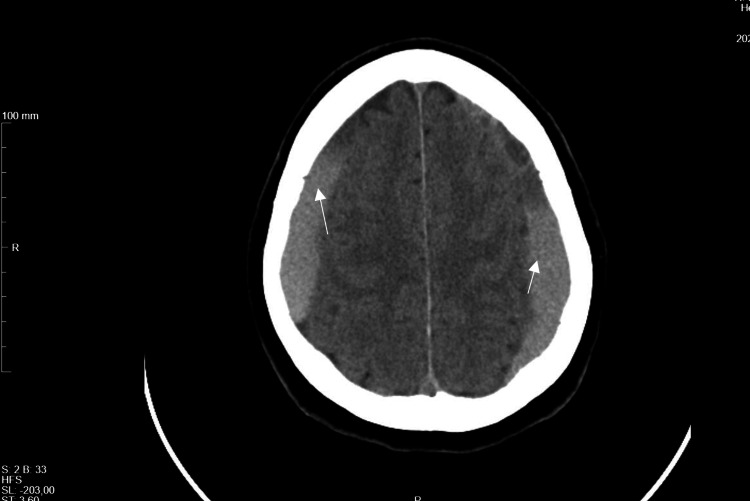
CT-scan showing bilateral frontoparietal hypodense subdural collections Arrows: subdural collections

The first brain CT scan performed showed bilateral frontoparietal hypodense subdural collections with approximate maximum thicknesses of 4 mm and diffuse cisternal and bilateral cortical sulcus effacement, suggesting the initial clinical hypothesis of cerebrospinal fluid hypotension (Figure 1). 

**Figure 2:**
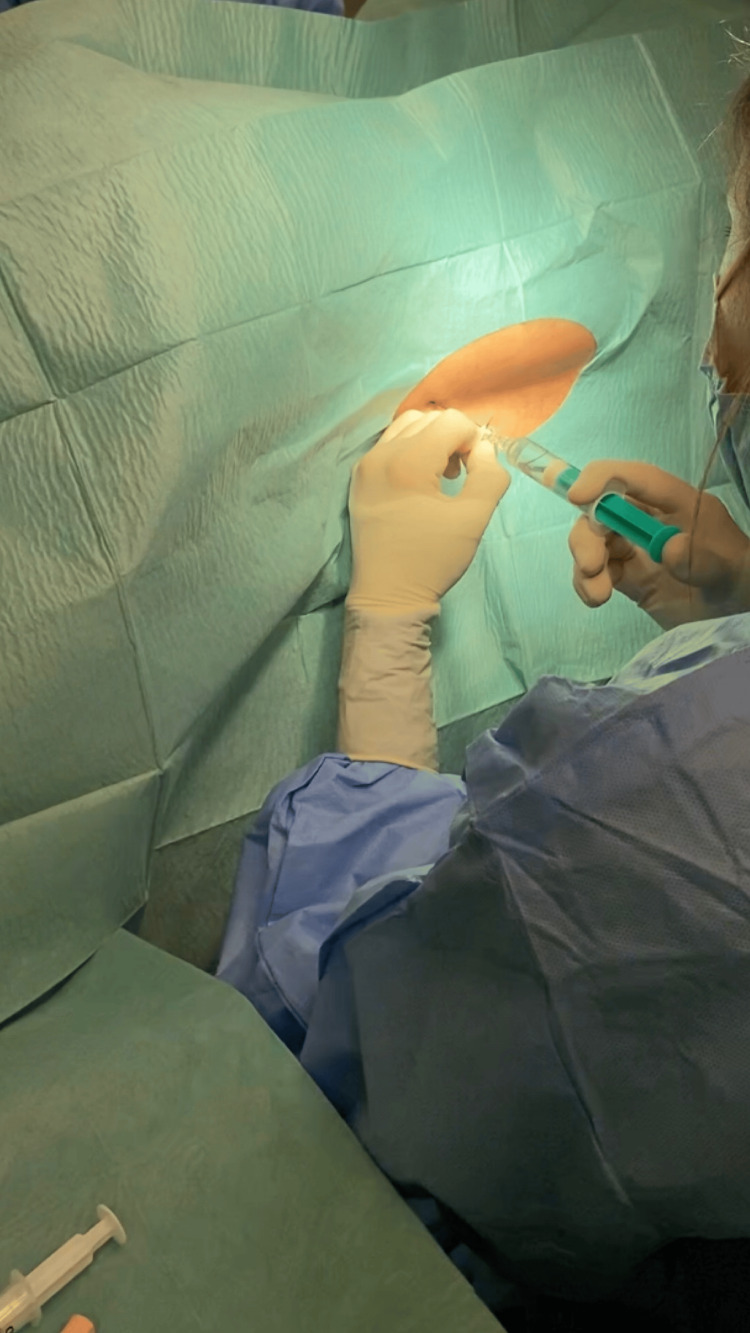
Reaching epidural space with an 18-gauge Tuohy needle

**Figure 3:**
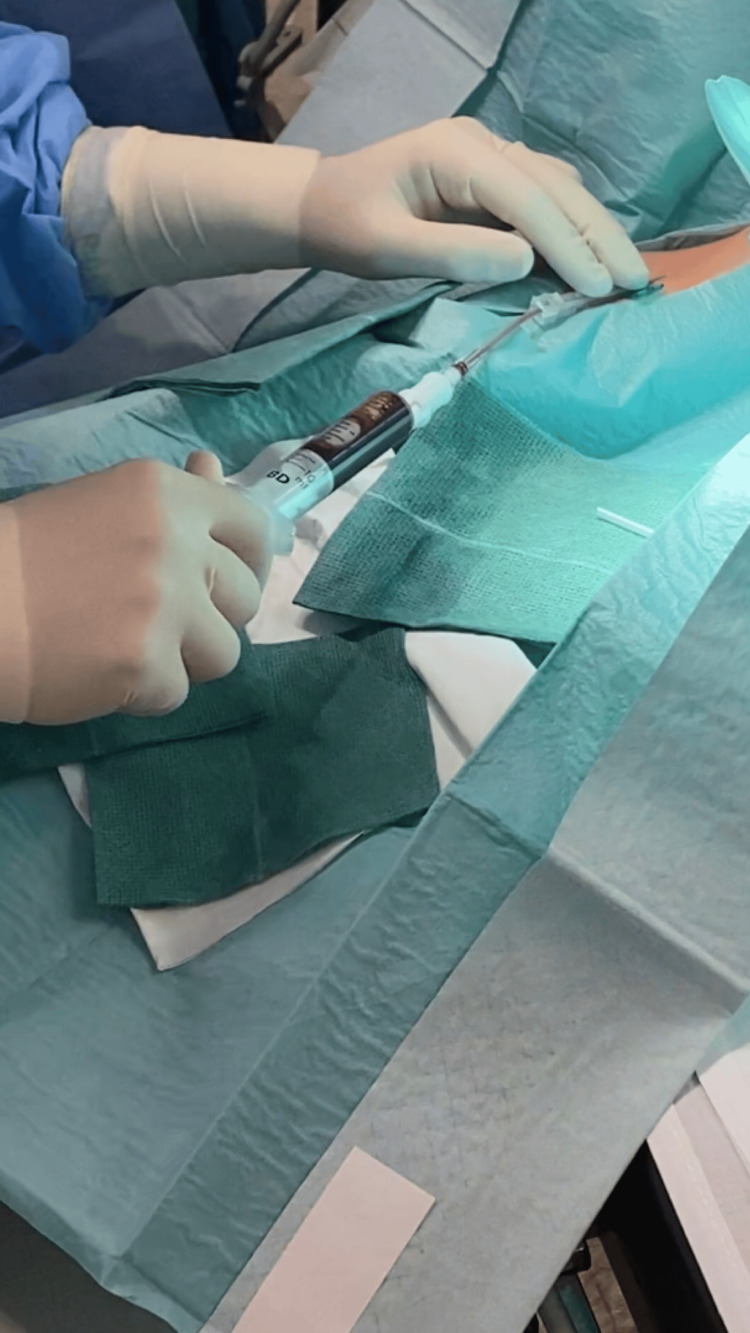
Collecting a blood sample from the patient’s right antecubital fossa

**Figure 4:**
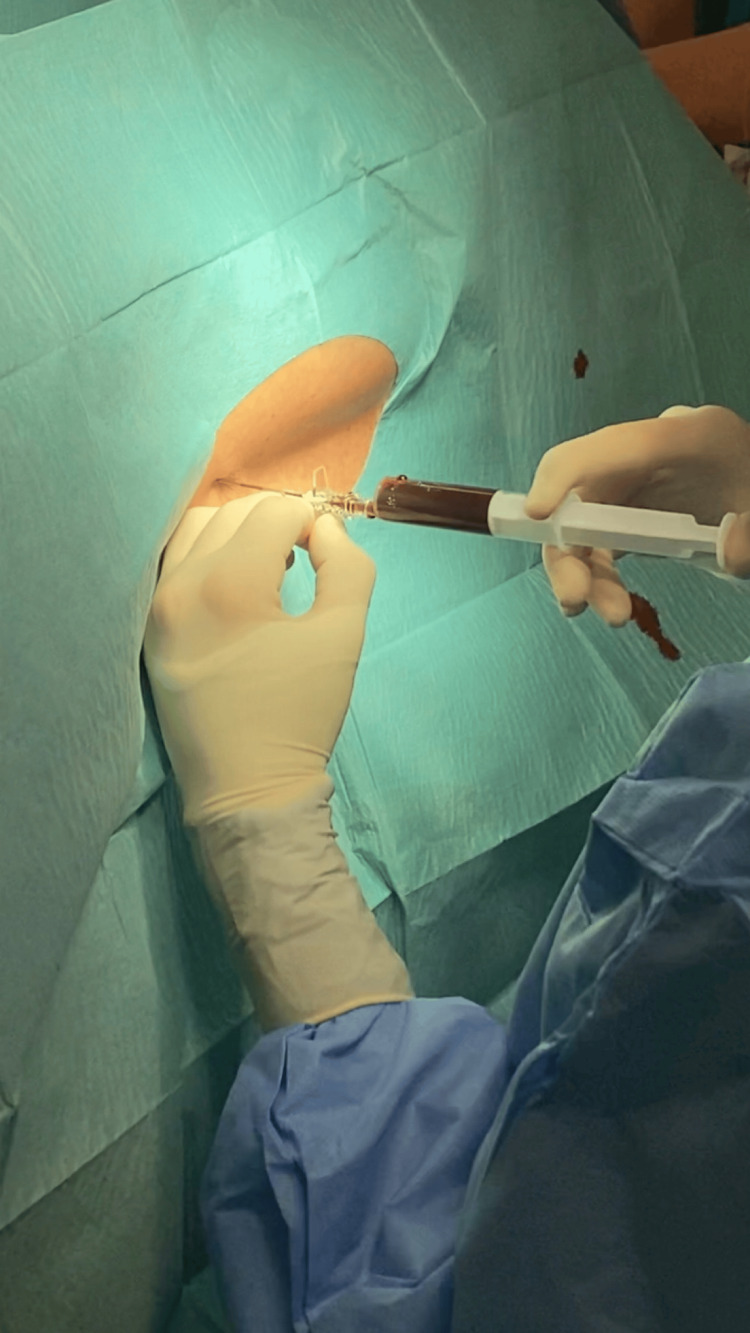
Administration of 15 mL of blood in the epidural space through an 18-gauge Tuohy needle

At this time, the patient refused hospital admission for treatment and returned a month later due to no clinical improvement. A new brain CT scan was performed, showing bilateral frontoparietal subdural hematomas with signs of recent bleeding of approximate maximum thicknesses of 16 mm. The patient was admitted to the ward with the diagnosis of SIH complicated with subdural hematomas and started conservative treatment with bed rest, paracetamol (1g every eight hours), caffeine, ergotamine (1mg SOS), and corticosteroid therapy with dexamethasone (4mg every eight hours). At this time, a spinal MRI and an MRI myelography revealed no CSF fistula sites. Two weeks later, a brain CT scan was performed and revealed a volume reduction of the subdural hematomas, with an approximate maximum thickness of 11 mm. Once there wasn’t a clinical improvement, an epidural blood patch (EBP) was proposed and accepted by the patient. The technique was performed in the operating room by the anesthesia team with the patient in the right lateral decubitus position, in L3-L4 space, and using an 18-gauge Tuohy needle (Figure 2). The blood sample was collected from the patient’s right antecubital fossa (Figure 3). A total of 15 mL of blood was administered in the epidural space. Both techniques were achieved under aseptic conditions (Figure 4).

**Figure 5:**
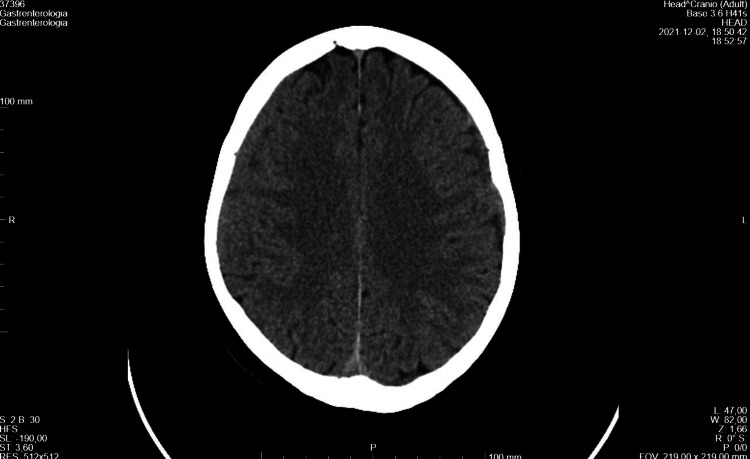
CT-scan with complete reabsorption of the bilateral subdural laminae and resolution of the previous findings.

Six hours later, there were no complaints of headaches, even with movement, sitting, or standing positions. Hospitalization was uneventful, and the patient was discharged four days after the procedure. At six and twelve months of follow-up, the patient was asymptomatic, with imaging exams showing complete reabsorption of the bilateral subdural laminae and resolution of the previous findings (Figure 5).

## Discussion

SIH is characterized by a debilitating orthostatic headache, low CSF pressure, and/or CSF leak without a previous history of head trauma or dural puncture [2-5].

SIH is an uncommon cause of headache, with an estimated prevalence and incidence of 1:50 000 and 2-5:100000 cases per year [2-5]. The exact pathophysiological mechanism is unknown; however, it is thought to result from singular or multiple leaks of CSF, often associated with an underlying connective tissue disorder that predisposes to weakness of the dura mater. Thoracic spine or cervicothoracic junction levels are the most frequent localizations of CSF leaks [2-4]. Women seem to be more affected than men, and it has a peak incidence around 30-50 years old, even though there are pediatric cases reported [2,6].

The most frequent symptom is a mild to severe bilateral, occipital, frontal, or diffuse orthostatic headache, making orthostatism and ambulation difficult. These postural headaches tend to worsen with the Valsalva maneuver and improve with the supine position. Other symptoms that could be associated with this condition are neck or intracapsular pain, nausea and vomiting, and auditory disturbance. Subdural hematomas (SDH) are also a complication of SIH. Typically, they are chronic, with or without an acute hemorrhagic component, well tolerated, and if the treatment of the underlying CSF leak is effective, the hematoma evacuation might be avoided [3]. Brain MRI abnormalities, particularly diffuse pachymeningeal enhancement, were detected in 73% of patients with SIH in a recent meta-analysis, but they were normal in about 20% of patients, so a normal MRI does not exclude SIH [2].

The treatment of choice as first-line for SIH is usually conservative, with hydration and bed rest. If the patient remains symptomatic or if an SDH appears on brain imaging, an epidural blood patch (EBP) should be considered to accelerate healing and avoid expansion of the SIH [2-6]. For that reason, some studies suggest performing a CT scan after 10 days of conservative therapy and performing an EBP within one to two weeks of refractory conservative treatment [3,5].

Although EBP is generally considered safe, there are some risks associated with this procedure. Potential complications include infection, bleeding, or a nerve injury. A thorough risk-benefit assessment is crucial before performing an EBP. Close neurological monitoring is essential after an EBP, especially when there is a subdural hematoma. Any signs of neurological deterioration, such as worsening headaches, changes in consciousness, or focal neurological deficits, should be promptly evaluated. Some experts even recommend delaying EBP until there is evidence of subdural hematoma stabilization or resolution to minimize the risk of exacerbating bleeding [2-6].

A recent systematic review concluded that although extradural CSF leak was only evident in 48% to 67% of the patients with an MRI, the first EBP was reported to be successful in 64% of the patients, without the need for further intervention, and also that similar results were found between target and nontargeted EBP. This systematic review also demonstrates that larger EBP (>20ml) had a higher success rate compared with smaller EBP [2].

## Conclusions

This case highlights the challenging nature of SIH and its potential complications. SIH remains an underdiagnosed condition due to its variable presentation and lack of awareness. This case emphasizes the importance of early recognition and appropriate management.

Conservative measures are the initial treatment for SIH. However, when conservative therapy proves ineffective or when complications like subdural hematomas arise, as seen in this case, an EBP can be a highly successful intervention. This case underscores the potential effectiveness of EBP in treating SIH and associated complications, offering a promising solution for patients who fail to respond to conservative management. This procedure seems to have a safe profile with minor transient adverse effects. Further research and awareness regarding SIH are needed to ensure this condition's timely diagnosis and effective management.
